# The time-fractional kinetic equation for the non-equilibrium processes

**DOI:** 10.1038/s41598-021-00135-7

**Published:** 2021-10-18

**Authors:** Ekrem Aydiner

**Affiliations:** Department of Physics, Faculty of Science, İstanbul University, 34134 Istanbul, Turkey

**Keywords:** Biological physics, Statistical physics, thermodynamics and nonlinear dynamics

## Abstract

In this study, we consider the non-Markovian dynamics of the generic non-equilibrium kinetic process. We summarize the generalized master equation, the continuous and discrete forms of the time-fractional diffusion equation. Using path integral formulation, we generalized the solutions of the Markovian system to the non-Markovian for the non-equilibrium kinetic processes. Then, we obtain the time-fractional kinetic equation for the non-equilibrium systems in terms of free energy. Finally, we introduce a time-fractional equation to analyse time evolution of the open probability for the deformed voltage-gated ion-channel system as an example.

## Introduction

A stochastic theory of the kinetics in univariant, non-equilibrium systems that undergoes phase transitions has been formulated in Refs.^1–5^. In these studies, the path integral formulation of the density distribution and conditional probability was given based on the Markovian master equation^6,7^ and the kinetic transition probability^8–10^.

Although the path integral approximation for the stochastic Markovian and non-Markovian processes are well known and well-studied topics in physics^11–16^, to the best of our knowledge, the path integral formulation of the non-Markovian dynamics of the non-equilibrium kinetic processes has never been discussed so far. Therefore, in this study, we generalized the path integral solutions of the Markovian systems to the non-Markovian case for the non-equilibrium kinetic system according to the methods presented in Refs.^1–5^.

Therefore, in this study, we consider a generic non-equilibrium kinetic system with non-Markovian dynamics. Following the formalism in Refs.^1–5^ we construct the conditional probability for the non-Markovian dynamics by using path integral formulation. Then, we show that the non-Markovian processes in the non-equilibrium system kinetic systems lead to time-fractional kinetic equations. Additionally, we consider the simplest voltage-gated ion-channel system as an example to analyze the time-fractional dynamics of the open probability.

The outline of our paper is given as follow: First, we introduce the non-Markovian master equation. Then, in the following section, we obtain the time-fractional kinetic equation for the non-Markovian dynamics of the non-equilibrium kinetic process. Here, we also introduce the fractional kinetic equation of the deformed voltage-gated ion-channel system. Finally, we review the obtained results and present a brief discussion.

## Generalized Master equation

First, we briefly give the information about the master equation for the non-Markovian dynamics which is called as generalized master equation (GME) in the literature^17–19^. The time evolution of finding probability *P*(*x*, *t*) can be presented on the one-dimensional space by

1 $$\begin{aligned} \frac{\partial P(x,t)}{\partial t } = \int _{-\infty }^{\infty } dx^{\prime } \int _{0}^{t} dt^{\prime } \mathfrak {R}(x,x^{\prime },t-t^{\prime }) P(x^{\prime },t^{\prime }), \end{aligned}$$

where $\mathfrak {R}(x,x^{\prime },t-t^{\prime })$ is the transfer probability kernel from the position *x* to $x^{\prime }$. As seen from Eq. (1) that GME is very different from Markovian form. Here, we use the probability function of the generalized master equation kernel $\mathfrak {R}(x,x^{\prime },t-t^{\prime })$ by reformulated the path integral formulation based on previous approximations^1–4^.

The Fourier-Laplace transformation of Eq. (1) is written as

2 $$\begin{aligned} uP(k,u) - W_{0} (k) = \mathfrak {R}(k,u) * P(k,u), \end{aligned}$$

where *u* is the Laplace variable, *k* is the wave number and $f(x)*g(x)\equiv \int _{-\infty }^{\infty } dx^{\prime }f(x-x^{\prime })g(x^{\prime })$ denotes a Fourier convolution of the *f* and *g* functions. Dividing by *u*, after Laplace inversion and differentiation $\frac{\partial }{\partial t}$ we obtain another representation

3 $$\begin{aligned} \frac{\partial P(x,t)}{\partial t } = \frac{\partial }{\partial t} \int _{-\infty }^{\infty } dx^{\prime } \int _{0}^{t} dt^{\prime } \tilde{\mathfrak {R}}(x,x^{\prime },t-t^{\prime }) P(x^{\prime },t^{\prime }), \end{aligned}$$

of the Eq. (1). The new kernel of the master equation is given by $\tilde{\mathfrak {R}}(x,x^{\prime };u)=\mathfrak {R}(x,x^{\prime };u)/u$, i.e. $\tilde{\mathfrak {R}}(x,x^{\prime };t)=\int _{0}^{t} dt \mathfrak {R}(x,x^{\prime };t)$ where $\tilde{\mathfrak {R}}$ can be presented with $\tilde{\mathfrak {R}}(x,x^{\prime };t) = W(x,x^{\prime }) \Pi (t)$. In this representation, the kernels $W(x,x^{\prime })$ and $\Pi$ are responsible for spatial correlations and memory in any stochastic process. These kernels are classified by the finite characteristic waiting time *T* and the finite jump length variance $\Sigma ^{2}$. It is known that, in the non-Markovian process, while the jump length variance $\Sigma ^{2}$ is finite, the waiting time *T* diverges due to the spatial deformations, entropic restrictions or other memory effects.

For a non-Markovian processes $\Pi (t)$ is represented by

4 $$\begin{aligned} \Pi (t) = \frac{1}{\Gamma (\gamma )} \left( \frac{t}{\tau }\right) ^{\gamma -1}, \end{aligned}$$

where $\Gamma$ is Gamma function, $\tau$ is the macroscopic relaxation parameter and the exponent $\gamma$ takes the value between $0<\gamma <1$. We note that Eq. (4) corresponds to the long-tailed waiting time probability distribution.

In the new situation, the new kernel is given by

5 $$\begin{aligned} \mathfrak {R}(x,x^{\prime };u) = W(x^{\prime },x) \frac{1}{\Gamma (\gamma )} \left( \frac{t}{\tau }\right) ^{\gamma -1}. \end{aligned}$$

The solution of the GME shows a strong dependence on its stochastic history. Therefore, the resulting equation is

6 $$\begin{aligned} \frac{\partial P(x,t)}{\partial t } = \frac{1}{\Gamma (\gamma )} \frac{\partial }{\partial t} \int _{0}^{t} dt^{\prime } \left( t-t^{\prime }\right) ^{\gamma -1} \int _{-\infty }^{\infty } dx^{\prime } W(x,x^{\prime }) P(x^{\prime },t^{\prime }). \end{aligned}$$

Equation (6) includes the defining expression^20^

7 $$\begin{aligned} _{0}D^{1-\gamma }_{t} P(x,t) = \frac{1}{\Gamma (\gamma )} \frac{\partial }{\partial t} \int _{0}^{t} dt^{\prime } \left( t-t^{\prime }\right) ^{\gamma -1} P(x,t), \end{aligned}$$

where $_{0}D^{1-\gamma }_{t}$ is the Riemann–Liouville fractional derivative^20^. Time-fractional master equation can be expressed in te form

8 $$\begin{aligned} \frac{\partial P(x,t)}{\partial t } = _{0}D^{1-\gamma }_{t} \int _{-\infty }^{\infty } dx^{\prime } W(x,x^{\prime }) P(x^{\prime },t). \end{aligned}$$

Here we briefly summarize that, the non-Markovian dynamics in the continuum limit leads to the time-fractional differential equation Eq. (8). The discrete form of Eq. (8) is given by

9 $$\begin{aligned} \frac{\partial P(x,t)}{\partial t } = _{0}D^{1-\gamma }_{t} \sum _{x^{\prime }} W(x,x^{\prime }) P(x,t^{\prime }). \end{aligned}$$

Using Eq. (9) we will construct the path integral formulation of the conditional probability function *P*(*x*, *t*).

## Time-fractional kinetic equation

It should be noted that the kernel of the spatial correlation for the kinetic processes can be defined in terms of Helmholtz free energy

10 $$\begin{aligned} W(x,x^{\prime })= \exp (-\frac{(x^{\prime } - x)^{2}}{\Delta }) \exp \{-\beta \frac{1}{2} [F(x^{\prime }) - F(x)], \end{aligned}$$

where $\beta$ is the inverse temperature, $\Delta$ is a constant and *F* is the free energy of the thermodynamic system. This definition of the kinetic transition probability in Eq. (10) suggested by Langer^8–10^, which is an extension of a model proposed by Glauber^21^ based on Zwanzig theory^22,23^.

Afterwards, following previous theoretical schema^1–5^ we construct the path integral definition of the probability function for the non-Markovian dynamics of the generic non-equilibrium kinetic. Thus, we write the master equation in Eq. (9) can be given in the form

11 $$\begin{aligned} \frac{\partial P(x,t)}{\partial t } = - _{0}D^{1-\gamma }_{t} H(x,\partial _{x})P(x,t), \end{aligned}$$

where $H(x,\partial _{x})$ is written as

12 $$\begin{aligned} H(x,\partial _{x}) P(x,t) = \sum _{\delta } (1 - e^{-\delta \partial /\partial x} ) W (x\rightarrow x+\delta ) P(x,t), \end{aligned}$$

which causes the Kramers-Moyal expansion^24,25^

13 $$\begin{aligned} H(x,\partial _{x}) P(x,t)= \sum _{m=1}^{\infty } \frac{(-1)^{m+1}}{m !} \delta ^{n} \frac{\partial ^{m}}{\partial x^{m}} \sum _{\delta } \delta ^{m} W (x\rightarrow x+\delta ) P(x,t), \end{aligned}$$

where $\frac{\partial ^{m}}{\partial x^{m}}$ operates at the same time both $W (x\rightarrow x+\delta ) P(x,t)$ and $W (x\rightarrow x+\delta )$. The sums are over all possible values of the multi-indices *m*. It should also be noted that we set $\delta = x^{\prime }-x$ and $x^{\prime }=x+\delta$ in Eqs. (12) and  (13) for the convenience.

At this point, by using definition of the derivatives we can arrange the left side of Eq. (11)^1–5^, and then we can write the probability function $P(x,t+\Delta t)$ as

14 $$\begin{aligned} P(x,t+\Delta t) = \{ 1 + \Delta t \ _{0}D^{1-\gamma }_{t} \sum _{m=1}^{\infty } \frac{(-1)^{m+1}}{m !} \delta ^{n} \frac{\partial ^{m}}{\partial x^{m}} \sum _{\delta } \delta ^{m} W (x\rightarrow x+\delta \} P(x,t). \end{aligned}$$

We define the Fourier transform $\mathcal {F} \{P\}(k,t+\Delta t)$ of Eq. (14) as

15 $$\begin{aligned} \mathcal {F} \{P\}(k,t+\Delta t) = \mathcal {F} \{P\}(k,t) + \Delta t \ _{0}D^{1-\gamma }_{t} \sum _{m=1}^{\infty } \frac{(-1)^{m+1}}{m !} \delta ^{m} k^{m} (-i)^{|m|} \} \mathcal {F} \{W P\}(k,t). \end{aligned}$$

This of course is represented by

16 $$\begin{aligned} \mathcal {F} \{P\}(k,t+\Delta t) = (2\pi )^{-1/2} \int _{-\infty }^{\infty } dx_{0} e^{ikx_{0}} \{ 1 + \Delta t \ _{0}D^{1-\gamma }_{t} H(x_{0},-ik) \} P(x_{0},t), \end{aligned}$$

where $H(x_{0},-ik)$ is obtained from Eq. (13) by replacing $\partial /\partial x$ with $-ik$ and *x* with $x_{0}$^2–4^. On the other hand, the inverse Fourier transform of Eq. (16) is given by

17 $$\begin{aligned} P(x,t+\Delta t) = (2\pi )^{-1/2} \int dk e^{-ikx} \mathcal {F} \{P\}(k,t+\Delta t). \end{aligned}$$

Here, introducing Eqs. (16) into (17) and recognizing that for small $\Delta t$ the curly bracket in Eq. (16) is an exponential, in this case, we obtain

18 $$\begin{aligned} P(x,t+\Delta t) = (2\pi )^{-m} \int _{-\infty }^{\infty } dx_{0} \int _{-\infty }^{\infty } dk \exp \\&\qquad \left[ - \Delta t \{ ik(x-x_{0})/ \Delta t \} - _{0}D^{1-\gamma }_{t} H(-ik, x_{0}) \right] P(x_{0},t). \end{aligned}$$

The kernel of Eq. (18) can be defined as

19 $$\begin{aligned} K_{\gamma }(x, t|x_{0},t) = \int \mathcal {D} (x_{0}) \int \mathcal {D}(k) \exp \left[ - \int _{t_{0}}^{t} dt^{\prime } \{ ik(t^{\prime }) \dot{x} (t^{\prime }) \} - _{0}D^{1-\gamma }_{t^{\prime }} H(-ik(t^{\prime }), x(t^{\prime }) \right] , \end{aligned}$$

where $\dot{x}(t^{\prime }) = (x-x_{0})/ \Delta t$. This kernel represents the path integral formulation of the conditional probability for non-Markovian kinetics. We clearly see that the integral argument in Eq. (19) corresponds to Lagrangian of the system, which is given as

20 $$\begin{aligned} L_{\gamma }(k, x, \dot{x}) = ik(t^{\prime }) \dot{x} (t^{\prime }) \} - _{0}D^{1-\gamma }_{t^{\prime }} H \left( -ik(t^{\prime }), x(t^{\prime }) \right) , \end{aligned}$$

where $H \left( -ik(t^{\prime }), x(t^{\prime }) \right)$ can be read as Hamiltonian. The path integral in Eq. (19) can be defined as the limit of the multiple integral

21 $$\begin{aligned}\int dx_{L-1}\ldots \int dx_{1} \int dk_{L-1} \ldots \int dk_{0} (2\pi )^{-mL} \exp \nonumber \\&\qquad \left[ -\Delta t \sum _{j=0}^{L-1} \{ \langle i k^{j}, (x^{j+1}-x^{j} ) / \Delta t \rangle - _{0}D^{1-\gamma }_{t^{\prime }} H(-ik^{j},x^{j} ) \} \right] , \end{aligned}$$

when $L=(t-t_{0}) / \Delta t \rightarrow \infty$. We consider here that the transition between the small paths along the trajectory are independent of each other. Now, by using Eq. (10) we can write $H(-ik,x)$ as

22 $$\begin{aligned} H \left( -ik, x) \right)= & {} i \sum _{j=1}^{m} k_{j} \int d\delta \delta _{j} \exp (-\Delta ^{-1} \langle \delta ,\delta \rangle ) \exp \left[ -\beta \frac{1}{2} \{ F(x+\delta ) - F(n) \} \right] - \frac{1}{2} \sum _{j=1}^{m} \sum _{i=1}^{m} k_{j} k_{i} \int d\delta \delta _{j} \delta _{i} \nonumber \\&\times \exp (-\Delta ^{-1} \langle \delta ,\delta \rangle ) \exp \left[ -\beta \frac{1}{2} \{ F(x+\delta ) - F(n) \} \right] . \end{aligned}$$

or very small $\Delta$ values, the integrations can be obtained as

23 $$\begin{aligned} H \left( -ik(t^{\prime }), x(t^{\prime }) \right) = - \frac{1}{2} \beta \Gamma \sum _{j=1}^{m} i k_{j} \frac{\partial F}{\partial x_{j}} - \frac{1}{2} \Gamma \sum _{j=1}^{m} k^{2}_{j}, \qquad \Gamma = \left( 2\pi \Delta \right) ^{m/2} \Delta . \end{aligned}$$

Introducing Eqs. (23) into (21) and integrating over *k*, we get the time-fractional kernel

24 $$\begin{aligned} K_{\gamma }(x,t|x_{0},t_{0}) = \int \mathcal {D} (x) \exp \left[ - \int _{t_{0}}^{t} dt^{\prime } \left\{ _{0}D^{\gamma }_{t} x (t^{\prime }) + \Gamma \beta \frac{\partial F(x(t^{\prime }))}{\partial x}\right \}^{2} \right] . \end{aligned}$$

The path of extreme probability are those for which $\int _{0}^{\tau }dt^{\prime } L_{\gamma }(x,\dot{x},k)$ is extremized, namely, time-fractional action integral satisfy the condition

25 $$\begin{aligned} \delta \int _{t_{0}}^{t} dt^{\prime } L_{\gamma } [k(t^{\prime }),x(t^{\prime }),\dot{x}(t^{\prime })] = 0, \end{aligned}$$

where $L_{\gamma }(k, x,\dot{x})$ is the time-fractional Lagrangian of the system. Euler-Lagrange equation in the action Eq. (25) can be solved due to *k* as

26 $$\begin{aligned} \frac{\partial L_{\gamma }}{\partial k} = 0. \end{aligned}$$

The solution of Eq. (26) gives the time-fractional kinetic equation

27 $$\begin{aligned} _{0}D^{\gamma }_{t} x (t) + \Gamma \beta \frac{\partial F(x(t))}{\partial x} = 0, \end{aligned}$$

where $0<\gamma <1$. As can be seen from Eq. (27) that the time evolution of the stochastic variable *x* is governed by integro-differential operator. The operator $_{0}D^{\gamma }_{t}$ points out that the solution of the kinetic equation Eq. (27) possesses Mittag–Leffler function.

### An example: the deformed ion-channel systems

In the simplest voltage-gated ion channel system, it is assumed that the channels are located on a two-dimensional membrane. At the equilibrium, the channels are open or closed depending on temperature and membrane voltage. Besides, the status of the channels depends on their intrinsic properties, the state of the channel is mainly determined by the membrane potential at a constant temperature. In these models, for simplicity, it is assumed that the channels are identical and distributed randomly on the membrane surface.

In this simple schema, the number of the channels is given by $n=n_{1}+n_{2}$ where $n_{1}$ denotes channels with energy $\varepsilon _{1}$ and $n_{2}$ admits the open channels with energy $\varepsilon _{2}$, respectively. In the statistical framework, the Helmholtz energy of such channel model around equilibrium can be written as^26,27^

28 $$\begin{aligned} F(x) = n \left[ (1-x) \varepsilon _{1} + x \varepsilon _{2} \right] + z e_{0} n (1-x) V - \beta ^{-1} \ln \Omega (x), \end{aligned}$$

where $x=n_{2}/n$ is the open probability $P_{0}$ under external potential *V*, $\Omega (x)$ corresponds to the number of configurations which is given by $\ln \Omega (x)= -n [(1-x) \ln (1-x) + x\ln x]$ and *z* is the number of charges, and $e_{0}$ is the charge of electron. It is shown that the open probability can be found from first derivative of the Helmholtz free energy in Eq. (28) as to the variable *x*. Thus it can be written as

29 $$\begin{aligned} P_{0} = x = \Big \{ 1 + \exp \left[ - \beta z e_{0} \left( V - V_{0} \right) \right] \Big \}^{-1}, \end{aligned}$$

where $V_{0} = - (\varepsilon _{1} - \varepsilon _{2}) / z e_{0}$ is the critical threshold voltage value. In the case of Markovian dynamics, it is assumed that the half of the channels on the membrane surface are open.

The open probability of the channel system in Eq. (29) is a well-known and well-studied topic in the literature. Furthermore, the kinetic behavior of the voltage gated ion channels was also examined within the framework of the Markovian formalism^26,27^. However, in the presence of the deformations such as a genetic mutation in the channel, we expect that ion channels have non-Markovian dynamics due to decoupling dynamics as stated in the previous section. As a result, channel conductivity is damaged and the cell may not be able to perform its previous tasks. If the channel kinetics is analyzed within the framework of the non-Markovian formalism, the time-fractional kinetic for the open probability of the ion channel system can be obtained as

30 $$\begin{aligned} _{0}D^{\gamma }_{t} P_{0} = \frac{\Gamma }{n} \Big \{ \beta z e_{0} (V-V_{0}) - \ln \frac{P_{0}}{1-P_{0}} \Big \}. \end{aligned}$$

As seen from this particular example, one can apply the fractional numerical integration method to Eq. (30) to obtain the time evolution of the open probability for the non-Markovian ion channel system far from steady-state. Hence, the time variation of the open probability and relaxation parameters of the non-Markovian ion channels can be obtained by using Eq. (30). The fractional derivative in Eq. (30) clearly indicates that the solution of the relaxation drastically deviates from the Markovian solution given in Refs.^26,27^.

## Conclusion

In this study, firstly, we briefly introduce the generalized master equation for non-Markovian dynamics. We also present the continuous and discrete forms of the time-fractional diffusion equation in the same section. In the subsequent section, we generalized the path integral solutions of the Markovian system to the non-Markovian for the non-equilibrium kinetic processes. Using path integral formulation we obtain the time-fractional kinetic equation for the non-equilibrium systems in terms of free energy. Here, we consider, as an example, a voltage-gated ion-channel system that behaves as a non-equilibrium system under cell voltage. We introduce the time-fractional kinetic equation for the open probability of the simple ion-channel system.

The non-Markovian dynamics and non-equilibrium behavior of the physical systems are very important two topics in physics. Indeed, many physical systems in nature may have one or both of these properties. Systems can be considered as systems which far from equilibrium that cannot reach equilibrium in very large time scales, and such systems can also be considered as systems that reach equilibrium in short time scales. On the other hand, we know that complex or disordered physical systems generally have non-Markovian dynamics. Therefore, it is very interesting to examine the time evolutions of kinetic systems with both non-equilibrium and Markovian dynamics.

The method presented in the study can be applied to the other physical systems to analyze the time-dependent evolution of the kinetic systems such as the interface dynamics of the phase transitions, kinetic flows, bacterial growth phenomena, other deformed ion channels, internet networks, and chemical kinetics^28–33^.
